# Identification of Hub Genes and Key Pathways Associated With Bipolar Disorder Based on Weighted Gene Co-expression Network Analysis

**DOI:** 10.3389/fphys.2019.01081

**Published:** 2019-08-20

**Authors:** Yang Liu, Hui-Yun Gu, Jie Zhu, Yu-Ming Niu, Chao Zhang, Guang-Ling Guo

**Affiliations:** ^1^Center for Evidence-Based Medicine and Clinical Research, Taihe Hospital, Hubei University of Medicine, Shiyan, China; ^2^Department of Orthopedic, Zhongnan Hospital of Wuhan University, Wuhan, China; ^3^Trade Union, Taihe Hospital, Hubei University of Medicine, Shiyan, China; ^4^Center of Women’s Health Sciences, Taihe Hospital, Hubei University of Medicine, Shiyan, China

**Keywords:** bipolar disorder, hub genes, WGCNA, pre-frontal cortex, modules

## Abstract

Bipolar disorder (BD) is a complex mental disorder with high mortality and disability rates worldwide; however, research on its pathogenesis and diagnostic methods remains limited. This study aimed to elucidate potential candidate hub genes and key pathways related to BD in a pre-frontal cortex sample. Raw gene expression profile files of GSE53987, including 36 samples, were obtained from the gene expression omnibus (GEO) database. After data pre-processing, 10,094 genes were selected for weighted gene co-expression network analysis (WGCNA). After dividing highly related genes into 19 modules, we found that the pink, midnight blue, and brown modules were highly correlated with BD. Functional annotation and pathway enrichment analysis for modules, which indicated some key pathways, were conducted based on the Enrichr database. One of the most remarkable significant pathways is the Hippo signaling pathway and its positive transcriptional regulation. Finally, 30 hub genes were identified in three modules. Hub genes with a high degree of connectivity in the PPI network are significantly enriched in positive regulation of transcription. In addition, the hub genes were validated based on another dataset (GSE12649). Taken together, the identification of these 30 hub genes and enrichment pathways might have important clinical implications for BD treatment and diagnosis.

## Introduction

Bipolar disorder (BD), like other mental illnesses, is considered a disease caused by abnormal development of the nervous system. During the neural development of patients, the ganglion birth and death rates increased (Uribe and Wix, 2012). According to the world health organization (WHO) statistics on BD, it is the sixth leading cause of disability worldwide.

The primary symptom of BD is a pathological increase or decrease in emotional activity, and the pathological process is a recurrent episode of mania and depression. The characteristics of mania are high mood, marked acceleration of thinking, and increased verbal movements. Conversely, depression often leads to low mood, slow thinking, reduced speech and movement, and loss of appetite (Cox et al., 2014). Thus, the alternation of these two diseases can significantly impact the health of the patient. BD can cause circadian rhythm disturbances (Gonzalez, 2014), cognitive impairment, verbal memory loss (Cardoso et al., 2015), and increase the risk of other diseases. Suicide (Calkin and Alda, 2012) is the leading cause of death in BD patients. In one long-term study, the average suicide rate of major emotional patients was 15–20%, and while BD patients had a rate of more than 20% (Pompili et al., 2013). Furthermore, the suicide of a patient can greatly affect their family and society.

Bipolar disorder has a high rate of misdiagnosis, prevalence and mortality. As the clinical manifestations of unipolar and bipolar depression are difficult to distinguish, and unipolar depression is more common (Hirschfeld, 2014). Therefore, it is crucial to find the hub genes that influence BD to improve the current treatment status. The prefrontal cortex is often called the center of the command and emotional control of the brain and is inextricably linked to mental illness. Microarray analysis of gene expression profiles in the prefrontal cortices from BD patients might contribute to the identification of hub genes and key pathways related to BD supporting the development of a new treatment strategy.

Weighted gene co-expression network analysis (WGCNA), which has been proposed by scholars Zhang and Horvath (2005), is a highly efficient and accurate biological methodology for Microarray data. The WGCNA package was released in 2008 (Langfelder and Horvath, 2008). To date, related studies have preformed gene expression microarray profiling of diseases to identify the hub genes related to diseases based on WGCNA, including osteoporosis (Farber, 2010), hepatocellular carcinoma (Yin et al., 2018), osteosarcoma (Liu et al., 2017), and renal cell carcinoma (Chen et al., 2018).

Weighted gene co-expression network analysis has been used to analyze large gene data sets. Build a matrix of all the genes and soft threshold of the data set is performed, and then the scale-free network is established through the soft threshold (Langfelder and Horvath, 2008). Tens of thousands of genes are divided into different modules in scale-free networks, and the genes in each module have the same expression pattern. After correlating these modules with the phenotypic characteristics of the sample, modules with a high correlation with the sample features are selected. Finally, hub genes with high connectivity in the module were identified. These genes play key roles in the characteristics and the development of the disease. Thus, the aim of the present study was to find the hub genes, key pathways, and potential molecular mechanisms of BD.

## Materials and Methods

### Data Collection and Pre-processing

The gene expression microarray profile of BD was obtained from the gene expression omnibus (GEO) database^1^ (Barrett et al., 2013). There were 205 samples in the data set of GSE53987 (Lanz et al., 2019), including 48 patients with schizophrenia, 52 patients with BD, 50 patients with major depressive disorder, and 55 healthy individuals. Each state is divided into the pre-frontal cortex, striatum, and hippocampus, depending on the tissue source. In this study, we selected 36 samples, including 17 BD and 19 healthy controls (HC). All samples were derived from Postmortem human sample’s pre-frontal cortex, and the BD group was indistinguishable from the HC group in mean age, postmortem interval, brain pH, RNA integrity number (RIN). All tissue samples were based on the platform of the Affymetrix Human Genome U133 Plus 2.0 Array. Robust multi-array averaging (RMA) algorithm was used to process the original file to ensure the comparability of gene expression profiles (Irizarry et al., 2003). The nsFilter algorithm was used to remove the probes that had little variation in the expression value, as well as the probes that did not have a corresponding gene ID (Yin et al., 2018).

### Co-expression Network Construction

Weighted gene co-expression network analysis package was necessary for the co-expression network construction (Langfelder and Horvath, 2008). First, the function goodSamplesgenes was used to remove unqualified genes and samples, and Z.k < −2.5 was excluded from subsequent studies. Then, function pickSoftThreshold was used to choose an appropriate soft-thresholding power (β) based on a scale-free topology criterion. The weighted adjacency matrix was constructed using the soft-thresholding power. The relationship between one gene and all other ones in the analysis was incorporated, and the adjacency matrix was transformed into the topological matrix (TOM) (Yip and Horvath, 2007). The genes demonstrated hierarchical clustering using the flashClust function, according to the TOM-based dissimilarity (1-TOM) measure. After hierarchical clustering, highly interconnected genes were assigned to the same module (Ravasz et al., 2002).

### Identification of Clinically Significant Modules and Functional Annotation

After the effective clinical information was imported into the co-expression network, the module eigengene (ME), gene significance (GS), and module membership (MM) were calculated. ME was representative of the gene expression profiles in a module, representing the average expression level of genes in this module. MM was defined as the degree of correlation between genes and module. If MM is close to 0, then the gene is not part of the module. Conversely, if MM is close to 1 or −1, the gene is highly correlated with the module. GS can be considered to be the association of individual genes with clinical information. The module could be a candidate if it has a high ME valve with the clinical trait (Langfelder and Horvath, 2008). The annotating the function of relevant modules was preformed to the potential mechanisms for the effects of corresponding clinical symptoms. All genes in the candidate modules were uploaded to the Enrichr database (Kuleshov et al., 2016) for pathway enrichment based on gene ontology (GO) functional annotation and the kyoto encyclopedia of genes and genomes (KEGG). GO enrichment analysis is primarily divided into three parts, molecular function (MF), biological process (BP), and cellular component (CC).

### Identification and Validation of Hub Gene

Hub genes are defined as genes with high correlation in candidate modules. High connectivity means that the connectivity ranked at top 10%. For example, if the module size was 1000, then the genes with top 100 were defined as the hub genes. Moreover, the hub gene has to meet the absolute value of the geneModuleMembership >0.80 and geneTraitSignificance >0.20. After identifying hub genes highly associated with clinical traits, search tool for the retrieval of interacting genes (STRING) (Szklarczyk et al., 2015) database was used to construct a PPI network and Cytoscape and (Shannon et al., 2003) visualize the PPI network. If a gene has high degrees in a PPI network, it will be defined as playing a critical role in the module. At last, hub genes selected will be validated. A separate dataset GSE12649 (Iwamoto et al., 2005) was used to verify the differential in the expression of hub genes in clinical traits using one-way ANOVA (*P* < 0.05). In order to prevent the gender and age of the sample having an impact on the selected hub genes, we used one-way covariance analysis to detect it.

## Results

### Data Pre-processing and Co-expression Network Construction

We downloaded 36 raw files from BD patients’ pre-frontal cortex from the GEO database. A total of 10094 genes were obtained from 36 samples through the RMA algorithm and nsFilter. In subsequent studies, samples GSM1304927 and GSM1304952 were filtered out (Supplementary Figure 1). On the basis of the scale-free topology criterion, to define the adjacency matrix, we selected β = 6 which the scale-free topology fit index reaches 0.88 as the soft-thresholding power (Supplementary Figure 2). As shown in Supplementary Figure 3, the 10,094 genes were divided into 19 modules based on the dynamic tree cutting method.

### Identification of Clinically Significant Modules and Functional Annotation

**FIGURE 1:**
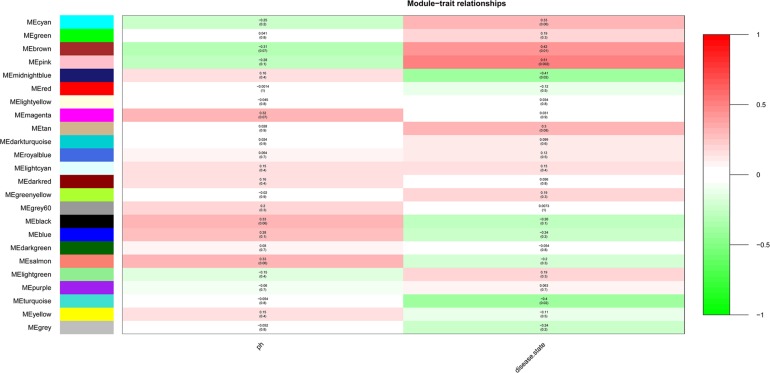
Module-trait relationships. Stage indicated bipolar disorder (BD) and non-BD.

**FIGURE 2:**
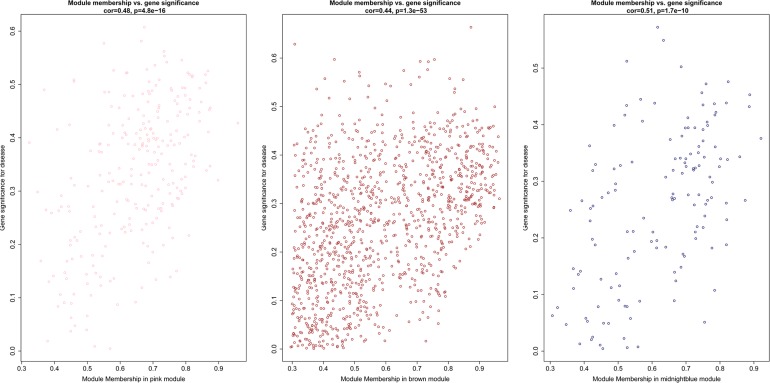
Scatter diagram for module membership vs. gene significance of stage (BD or non-BD) in three modules.

After the clinical traits of the samples were introduced into the weighted network (Figure 1), The study found Pink (*r* = 0.51, *P* = 0.002) module with 221 genes, brown (*r* = 0.42, *P* = 0.01) with 1104 genes, and midnightblue module (*r* = −0.41, *P* = 0.02) with 138 genes were highly correlated with the disease. In the following analysis, the GS and MM of the midnight blue module (cor = 0.51, *P* = 1.7e-10, Figure 2), the brown module (cor = 0.44, *P* = 1.3e-53, Figure 2) and the pink module (cor = 0.48, *P* = 4.8e-16, Figure 2) were calculated. In order to clarify the functional mechanism of targeted modules and diseases, 143 hub genes were uploaded to the Enrichr database for functional annotation. Supplementary Table 1 shows clear information about 143 hub genes. GO function annotation indicates that hub genes were enriched in eye development, G-protein coupled receptor complex, positive regulation of transcription, negative regulation of canonical Wnt signaling pathway, cytokine receptor activity complex, activating transcription factor binding, phosphatidylinositol binding, and others (*P* < 0.01) (Supplementary Table 2). Supplementary Table 3 shows that hub genes were enriched in the Hippo signaling pathway, thyroid hormone signaling pathway, signaling pathways regulating stem cell pluripotency, endocytosis, adherens junction, and others, based on KEGG pathway analysis (*P* < 0.01).

### Validation of Hub Gene and PPI Network Construction

**FIGURE 3:**
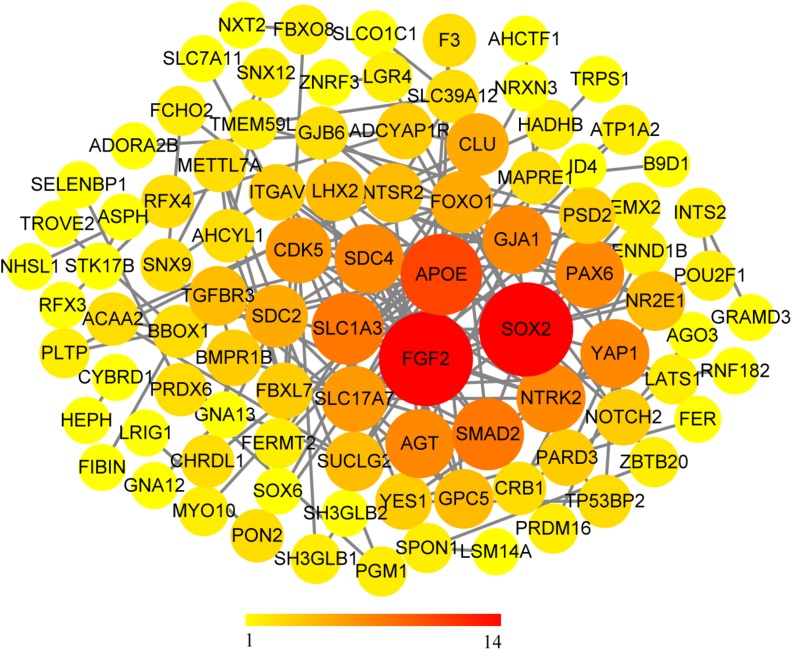
PPI network for all candidate modules.

**FIGURE 4:**
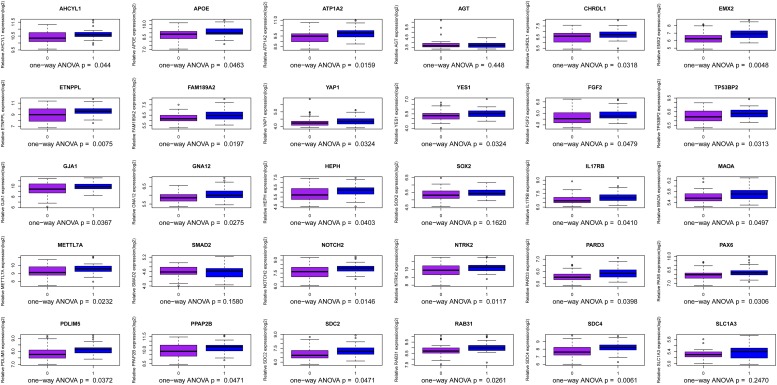
Boxplot for identification of hub genes in the three modules.

We use the methods mentioned above to identify hub genes. 143 hub genes in the three modules were identified. Figure 3 shows the PPI network for modules which are a candidate. The module network contained 96 nodes and 160 edges, three of which were identified as the high degree genes. Moreover, we found that hub genes with better connectivity in the PPI network are significantly enriched in the positive regulation of transcription (DNA-templated), including SOX2, FGF2, SMAD2, YAP1, PAX6, AGT, and FOXO1. This enrichment pathway might be a potential BD mechanism. All hub genes were validated based on GSE12649. Finally, we identified 30 hub genes common to both data sets as our final hub genes. Boxplots were used to show the validation results for 30 hub genes (Figure 4). Table 1 shows final hub genes in the three modules, and the covariate analyses of final hub genes are described in Supplementary Table 4.

**TABLE 1:** Hub genes from the interesting modules.

**Gene symbol**	**ENTREZ_GENE_ID**	**Gene ontology**	**geneModuleMembership**	**Gene trait significance**	**Up or down**
AGT	183	Alkylguanine DNA binding	0.887157454	0.298842057	Down
AHCYL1	10768	Adenosylmethionine hydrolase activity	0.892921034	0.263587391	Down
APOE	348	Cognition	0.906508824	0.259473598	Down
ATP1A2	477	Temperature gated cation channel activity	0.944732073	0.358469425	Down
CHRDL1	91851	Negative regulation of secondary growth	0.863182953	0.462817741	Down
EMX2	2018	Forebrain neuroblast differentiation	0.943545349	0.339285953	Down
ETNPPL	64850	/	0.905878301	0.312454124	Down
FAM189A2	9413	Gene expression	0.872771128	0.313792924	Down
FGF2	2247	fibroblast proliferation	0.9348346	0.45704696	Down
GJA1	2697	Gap junction	0.920113458	0.328482543	Down
GNA12	2768	Spermatid nucleus differentiation	0.867055073	0.311316484	Down
HEPH	9843	Copper exporting atpase activity	0.959262811	0.384839787	Down
IL17RB	55540	Cardiac fibroblast cell fate commitment	0.87323481	0.315545635	Down
MAOA	4128	Monoamine oxidase activity	0.889016805	0.495684	Down
METTL7A	25840	RNA polymerase II regulatory region DNA binding	0.93466073	0.322509631	Down
NOTCH2	4853	Notch signaling pathway	0.952637565	0.360026782	Down
NTRK2	4915	Regulation of neurotrophin TRK receptor signaling pathway	0.947485184	0.327326464	Down
PARD3	56288	Positive regulation of JAK STAT cascade	0.877332438	0.357811988	Down
PAX6	5080	Eye development	0.912624234	0.408751454	Down
PDLIM5	10611	Activation of bipolar cell growth	0.92796358	0.456391993	Down
PPAP2B	8613	Cell migration involved in coronary angiogenesis	0.882924502	0.299775962	Down
RAB31	11031	ER body	0.908780797	0.376400336	Down
SDC2	6383	Positive regulation of synapse maturation	0.935916785	0.333198546	Down
SDC4	6385	Small molecule catabolic process	0.889206651	0.361762932	Down
SLC1A3	6507	Negative regulation of L glutamate transport	0.930712467	0.322178332	Down
SMAD2	4087	SMAD2 protein complex	0.841744744	0.431128371	Down
SOX2	6657	Stem cell differentiation	0.960030288	0.437884345	Down
TP53BP2	7159	P53 binding	0.916365941	0.261334186	Down
YAP1	10413	Hippo signaling	0.946460117	0.317467764	Down
YES1	7525	Regulation of B cell antigen processing and presentation	0.913582422	0.40331199	Down

## Discussion

Bipolar disorder, a disease that is spread worldwide, has high rates of disability (Vos et al., 2012), mortality, and suicide, which can impose a significant burden on society (Adam, 2008). Therefore, exploring the hub genes and key pathways related to BD is essential for the diagnosis and treatment of patients with BD.

The weighted gene co-expression network analysis algorithm searches for effective information from gene expression microarray profile by building gene modules, and it tries to explain the significance of a gene module from the perspective of biology. In this study, we found that the pink, midnightblue, and brown modules were highly correlated with BD or no-BD. The expression of 30 genes in these three modules showed significant changes in patients with BD and non-BD individuals, 25 of which were validated through one-way ANOVA. Although other five genes have not been validated, most of them have been mentioned in previous studies (Meira-Lima et al., 2000; Medina et al., 2013; Ferensztajn-Rochowiak et al., 2016). These hub genes may have important clinical significance in the course of BD patient diagnosis and prognosis. After covariate analysis of all hub genes, we found that gender had little effect on these genes (*P* > 0.05). A few hub genes are affected by age factors (*P* > 0.05), including SCD4, PDLIM5, FAM189A2, and MAOA.

Kyoto encyclopedia of genes and genomes enrichment analysis suggested that the hub gene is mainly enriched in the Hippo signaling pathway, thyroid hormone signaling pathway, adherens junction, endocytosis, cell adhesion molecule, and mineral absorption. Meanwhile, we found that SMAD2 (SMAD family member 2), a member of the SMAD family, appears in most enrichment pathways. SMAD proteins are signal transducers and transcriptional modulators that mediate multiple signaling pathways. SMAD proteins regulate the signaling of transforming growth factor (TGF)-beta, and it plays a vital role in the growth, repair, and rotation of the nervous system (Liang et al., 2008).

The primary role of the Hippo signaling pathway is to regulate cell growth and apoptosis, maintaining organ size (Zhang et al., 2009). Moreover, it has another role in the establishment and maintenance of dendritic fields, since abnormalities in the Hippo signaling pathway can cause defects in these functions (Emoto, 2012). There is a significant difference in the dendritic structure between BD patients and normal individuals (Konopaske et al., 2014). The hub gene YAP1 (Yes-associated protein 1) is the main effector downstream of the Hippo signal pathway (Mizuno et al., 2012). Abnormalities in YAP1 expression will affect the function of the Hippo signal pathway. Also, YAP1 plays an essential role in the maintenance of neural progenitor cells (Mizuno et al., 2012). Neural progenitor cells regulate the production of the cerebral cortex (Arai and Taverna, 2017). The hub gene PARD3 (par-3 family cell polarity regulator) and Hippo signaling pathway together maintain ordered RGP (radial glial progenitor) splitting behavior and neuron generation in the mammalian cortex, and asymmetric division of RGPs regulates the generation of cortical neurons (Kodaka and Hata, 2015; Liu et al., 2018). Dysfunction of this mechanism often leads to cortical neuron damage and may lead to the development of BD. Moreover, a recent study also mentioned that BD is associated with the Hippo signaling pathway (O’Connell et al., 2018).

Gene ontology function annotation indicates that hub genes are significantly represented in some biological processes such as eye development, G-protein coupled receptor complex, positive regulation of transcription, negative regulation of canonical Wnt signaling pathway, cytokine receptor activity, and activating transcription factor binding. The Wnt signaling pathway contributes to the development of neural circuits and neuronal plasticity as well as early neural induction (Kiecker and Niehrs, 2001; Stern, 2001; Ciani and Salinas, 2005). Neurodevelopmental disorders are often one of the main pathological features of mental illness. There is already a large body of evidence confirming that abnormalities in the Wnt signaling pathway contribute to BD pathogenesis (Mulligan and Cheyette, 2017). We have obtained many biological processes through GO enrichment analysis, and although many of them have not been mentioned, these may be potential mechanisms underlying the development of BD.

For the PPI network, we found significant connectivity between the three genes, including SOX2 (SRY-box 2), FGF2 (fibroblast growth factor 2), and APOE (apolipoprotein E). SOX2 is a transcription factor that regulates stem cell proliferation and differentiation in the central nervous system (Phi et al., 2007; Koike et al., 2014). Moreover, the SOX2-encoded product SOX2ot is considered to be a potential marker of neurodegenerative diseases (Arisi et al., 2011). FGF2 plays a crucial role in maintaining differentiation and function of the central nervous system (Woodbury and Ikezu, 2014), and the level of FGF2 in patients with BD is higher than that in healthy people (Liu et al., 2014). NOTCH2 (notch receptor 2) plays a key role in the development of the nervous system (Sundararajan et al., 2018). A series of pathological and behavioral changes in BD patients may indicate that the development of the nervous system has been destroyed (O’Shea and McInnis, 2016). FGF2 can inhibit neuronal differentiation through the NOTCH pathway (Faux et al., 2001), which may affect the normal development of the nervous system, leading to BD. APOE apolipoprotein plays a key role in maintaining cerebral phospholipid homeostasis (Zhu et al., 2015) and plays a vital role in the pathogenesis and pathology of Alzheimer’s disease (AD) (Raber et al., 2004). SLC1A3 (solute carrier family 1 member 3) plays a role in the termination of excitatory neurotransmission in the central nervous system. SLC1A3 has been shown to decrease in BD patients (Medina et al., 2013). NTRK2 (neurotrophic receptor tyrosine kinase 2) is thought to have the effect of a mood stabilizer, which may affect the treatment of BD (Fabbri and Serretti, 2016). It is a receptor for neurotrophin brain-derived neurotrophic factor (BDNF). BDNF has nutritional and protective effects on nerves and is a potential biomarker for BD (Shaltiel et al., 2007; Munkholm et al., 2016). NTRK2 is considered a candidate gene for BD (Smith et al., 2009). AGT (angiotensinogen) was found to be associated with increased susceptibility to BD in Brazil (Meira-Lima et al., 2000).

Coincidentally, many genes with high connectivity in the PPI network were enriched in the positive regulation of transcription (DNA-templated), including SOX2, FGF2, SMAD2, YAP1, PAX6, AGT, and FOXO1. In a recent study, BD IPSC (induced pluripotent stem cells) is significantly different from normal IPSC in gene expression profiling, especially membrane receptor and ion channel genes, and calcium ion-related transcripts (Chen et al., 2014). It is well known that miRNA is one of the products of the transcription process, and miRNA expression level is closely related to transcriptional regulation. The expression levels of multiple MIRNAs in the prefrontal cortex of BD patients have changed (Kim A.H. et al., 2010; Moreau et al., 2011). The expression profile of astrocytes in the cerebral cortex of patients with BD has also increased (Toker et al., 2018). The activity of the transcription factor plays an essential role in neuronal function. Abnormal gene expression of transcription factors can affect the development and function of the nervous system and may lead to mental diseases, including BD (Pinacho et al., 2015).

Gap junction protein alpha 1 (GJA1) is a member of the connexin gene family, encodes connexin (Cx) proteins, Cx43 (De Bock et al., 2013). GJA1 is widely expressed in astrocytes, mice lacking GJA1 are grossly normal, but their synaptic plasticity is reduced (Han et al., 2014). The development of BD also leads to changes in synaptic plasticity (Berridge, 2014), and BD pathology is associated with apoptosis and synaptic dysfunction (Kim H.W. et al., 2010). Moreover, the traditional therapeutic drug lithium agent of BD has a potential effect on the activation of neural plastic channels during the treatment process (Machado-Vieira, 2018). However, there is currently no evidence that BD is associated with GJA1.

Both Bipolar disorder and Alzheimer’s disease are neurologically related diseases, one is neurodevelopmental disorder (Sanches et al., 2008), and the other is neurodegeneration (Hogh, 2017). But they have related pathophysiological processes (Maddison and Giorgini, 2015), and some of the disease-causing genes are overlapping (Drange et al., 2019). In our results, 16 of the 30 hub genes are related to AZ. Among them, AGT, APOE, GJA1, MAOA, RAB31, NOTCH2, PDLIM5, SLC1A3, SMAD2, and YAP1 are biomarkers or related risk factors of AZ (Wu et al., 2004; Ueberham et al., 2009; Kanai et al., 2013; Cheng et al., 2018; Kajiwara et al., 2018; Liang et al., 2018; Quartey et al., 2018; Xu et al., 2018; Zhao et al., 2018). This further proves that there may be a certain relationship between BD and AZ, with overlapping mutant genes.

To the best of our knowledge, this is the first WGCNA analysis of RNA data from tissues of the prefrontal cortex of BD patients. The primary advantage of this study is to combine the gene expression data of BD patients with clinical traits. By choosing the appropriate weighting coefficient to weight the correlation coefficient between genes, the WGCNA algorithm could make the gene network obey the scale-free network distribution and divide the genes with similar expression into the same module. Then, the modules with high correlation with traits were selected, and hub genes in the modules were identified. Finally, the potential relationship between modules and hub genes and traits was further discussed through gene enrichment analysis.

Our research has some limitations. Compared to previous WGCNA analyses of other disease, our sample size was insufficient, and there might be some bias. Nevertheless, we were able to replicate many published findings successfully, and we used a separate database to verify the experimental results. Although most hub genes were verified, a few hub genes were not, probably because the samples of GSM12649 were not from the same platform. Another limitation is that similar studies have been published. However, we have discovered many new hub genes and enrichment pathways. Future studies will involve more experiments to prove and explain how hub genes and hub pathways affect BD development.

## Conclusion

Our study based on WGCNA analyses found the 30 hub genes in three modules related to BD, and the Hippo signaling pathway and positive regulation of transcription may be one of the potential BD mechanisms. These hub genes and enrichment pathways may have important clinical implications for BD treatment and diagnosis.

## Data Availability

Publicly available datasets were analyzed in this study. This data can be found at: https://www.ncbi.nlm.nih.gov/geo/query/acc.cgi?acc=GSE53987.

## Author Contributions

YL, CZ, and Y-MN conceived and designed the study. YL and CZ performed the analysis. YL, H-YG, and CZ analyzed the results. G-LG, JZ, and Y-MN contributed analysis tools. G-LG and CZ contributed to the writing of the manuscript. G-LG contributed to the revision of this research, especially in the design of “ranked at top 10%” and “GS>0.2, as the routine boundary value,” and language modification. All authors reviewed the manuscript.

## Conflict of Interest Statement

The authors declare that the research was conducted in the absence of any commercial or financial relationships that could be construed as a potential conflict of interest.

The reviewer X-TZ declared a shared affiliation, with no collaboration, with one of the authors H-YG to the handling Editor at the time of review.

## Footnotes

1 http://www.ncbi.nlm.nih.gov/geo
