# Supplementary material for: Identification of Hub Genes and Key Pathways Associated With Bipolar Disorder Based on Weighted Gene Co-expression Network Analysis
Source: Front Physiol. 2019 Aug 20;10:1081. doi: 10.3389/fphys.2019.01081 (PMC6710482; doi:10.3389/fphys.2019.01081)
Supplement: Supplementary file 1 [file Data_Sheet_1.doc]

**Legends of Supplement Figure and Table**

**Supplementary Table 1** shows information about 143 hub genes

**Supplementary Table 2** The GO analysis of the three modules

**Supplementary Table 3** The KEGG pathway analysis of the three modules

**Supplementary Table 4** Covariance analysis of all key genes (Gender and Age)

**Supplementary Figure 1** Sample dendrogram and trait heatmap

**Supplementary Figure 2** Analysis of network topology for various soft-theholding powers

**Note:** The left panel showed the scale-free fit index, signed R^2 (y-axis) and the soft threshold power (x-axis).

**Supplementary Figure 3** Clustering dendrogram of genes

**Supplementary Table 1 shows information about 143 hub genes**

| **Gene Symbol** | **ENTREZ_GENE_ID** | **GeneModuleMembership** | **GeneTraitSignificance** | **MODULE** |
| --- | --- | --- | --- | --- |
| PON2 | 5445 | 0.963712324 | 0.310002791 | brown |
| SOX2 | 6657 | 0.960030288 | 0.437884345 | brown |
| HEPH | 9843 | 0.959262811 | 0.384839787 | brown |
| ARHGEF26 | 26084 | 0.953638938 | 0.407914262 | brown |
| NOTCH2 | 4853 | 0.952637565 | 0.360026782 | brown |
| SPON1 | 10418 | 0.948668886 | 0.499623701 | brown |
| BMPR1B | 658 | 0.947868426 | 0.388972262 | brown |
| NTRK2 | 4915 | 0.947485184 | 0.327326464 | brown |
| YAP1 | 10413 | 0.946460117 | 0.317467764 | brown |
| NHSL1 | 57224 | 0.946117465 | 0.29252537 | brown |
| MYO10 | 4651 | 0.945587444 | 0.291355367 | brown |
| ATP1A2 | 477 | 0.944732073 | 0.358469425 | brown |
| NPAS3 | 64067 | 0.944167864 | 0.459683747 | brown |
| EMX2 | 2018 | 0.943545349 | 0.339285953 | brown |
| GRAMD3 | 65983 | 0.942353294 | 0.416359734 | brown |
| SLC7A11 | 23657 | 0.940577612 | 0.355679154 | brown |
| ZNRF3 | 84133 | 0.937808549 | 0.378971005 | brown |
| SDC2 | 6383 | 0.935916785 | 0.333198546 | brown |
| FGF2 | 2247 | 0.9348346 | 0.45704696 | brown |
| METTL7A | 25840 | 0.93466073 | 0.322509631 | brown |
| RFX4 | 5992 | 0.934249039 | 0.37537662 | brown |
| SSPN | 8082 | 0.932667802 | 0.418091573 | brown |
| ASPH | 444 | 0.931001986 | 0.4548887 | brown |
| SLC1A3 | 6507 | 0.930712467 | 0.322178332 | brown |
| PDLIM5 | 10611 | 0.92796358 | 0.456391993 | brown |
| GPR125 | 166647 | 0.926419727 | 0.39238347 | brown |
| APCDD1 | 147495 | 0.925840698 | 0.400920328 | brown |
| CLU | 1191 | 0.924522503 | 0.326695766 | brown |
| GJA1 | 2697 | 0.920113458 | 0.328482543 | brown |
| BBOX1 | 8424 | 0.919287513 | 0.338359724 | brown |
| NR2E1 | 7101 | 0.917897428 | 0.352022431 | brown |
| TP53BP2 | 7159 | 0.916365941 | 0.261334186 | brown |
| YES1 | 7525 | 0.913582422 | 0.40331199 | brown |
| PAX6 | 5080 | 0.912624234 | 0.408751454 | brown |
| TGFBR3 | 7049 | 0.911738219 | 0.439565219 | brown |
| HADHB | 3032 | 0.910276234 | 0.410393769 | brown |
| ZBTB20 | 26137 | 0.909911945 | 0.422677096 | brown |
| FBXL7 | 23194 | 0.909073836 | 0.365398613 | brown |
| RAB31 | 11031 | 0.908780797 | 0.376400336 | brown |
| SASH1 | 23328 | 0.908612387 | 0.364370202 | brown |
| PLSCR4 | 57088 | 0.908533552 | 0.417094439 | brown |
| GPAM | 57678 | 0.90849189 | 0.374461177 | brown |
| DPY19L3 | 147991 | 0.907488987 | 0.455808598 | brown |
| APOE | 348 | 0.906508824 | 0.259473598 | brown |
| CLDN10 | 9071 | 0.906478447 | 0.26780701 | brown |
| LRP4 | 4038 | 0.906376586 | 0.330720741 | brown |
| ETNPPL | 64850 | 0.905878301 | 0.312454124 | brown |
| UG0898H09 | 643763 | 0.904422781 | 0.339045273 | brown |
| GPC5 | 2262 | 0.903876136 | 0.360982557 | brown |
| LRIG1 | 26018 | 0.90332224 | 0.294667273 | brown |
| PLTP | 5360 | 0.902380729 | 0.298919048 | brown |
| ADORA2B | 136 | 0.902380141 | 0.274060747 | brown |
| MLC1 | 23209 | 0.901191748 | 0.235594412 | brown |
| CRB1 | 23418 | 0.900235319 | 0.451274904 | brown |
| ACAA2 | 10449 | 0.900179982 | 0.379677956 | brown |
| SLC16A9 | 220963 | 0.900114331 | 0.408863797 | brown |
| FERMT2 | 10979 | 0.900013413 | 0.378236869 | brown |
| FIBIN | 387758 | 0.899652963 | 0.422726125 | brown |
| EPHX1 | 2052 | 0.899429918 | 0.296677447 | brown |
| PSD2 | 84249 | 0.898957989 | 0.307029152 | brown |
| FOXO1 | 2308 | 0.898207692 | 0.32746488 | brown |
| PRDX6 | 9588 | 0.89611816 | 0.226091496 | brown |
| SLC39A12 | 221074 | 0.895386115 | 0.293462946 | brown |
| NXT2 | 55916 | 0.893520158 | 0.446997459 | brown |
| AHCYL1 | 10768 | 0.892921034 | 0.263587391 | brown |
| SLC2A10 | 81031 | 0.892869919 | 0.326592735 | brown |
| SLC18B1 | 116843 | 0.892056671 | 0.412470678 | brown |
| MTM1 | 4534 | 0.892012312 | 0.472146899 | brown |
| C16orf89 | 146556 | 0.890076044 | 0.383051801 | brown |
| CYBRD1 | 79901 | 0.889429982 | 0.275312665 | brown |
| SDC4 | 6385 | 0.889206651 | 0.361762932 | brown |
| MAOA | 4128 | 0.889016805 | 0.495684 | brown |
| ITGAV | 3685 | 0.888244719 | 0.373943131 | brown |
| AGT | 183 | 0.887157454 | 0.298842057 | brown |
| ID4 | 3400 | 0.886831098 | 0.455663868 | brown |
| SOX6 | 55553 | 0.88656548 | 0.356731941 | brown |
| PRDM16 | 63976 | 0.886153313 | 0.258208214 | brown |
| GPM6B | 2824 | 0.88601583 | 0.301115789 | brown |
| NTSR2 | 23620 | 0.884188685 | 0.341280981 | brown |
| PPAP2B | 8613 | 0.882924502 | 0.299775962 | brown |
| LGR4 | 55366 | 0.880241123 | 0.417966263 | brown |
| TRPS1 | 7227 | 0.879990231 | 0.329440597 | brown |
| MAPRE1 | 22919 | 0.878668116 | 0.470500029 | brown |
| HTRA1 | 5654 | 0.87745464 | 0.298941559 | brown |
| F3 | 2152 | 0.877445767 | 0.377240204 | brown |
| PARD3 | 56288 | 0.877332438 | 0.357811988 | brown |
| PGM1 | 5236 | 0.877072538 | 0.366016318 | brown |
| SUCLG2 | 8801 | 0.876899203 | 0.318467557 | brown |
| GNA13 | 10672 | 0.875473984 | 0.387211743 | brown |
| MIR3682 | 100500850 | 0.874347568 | 0.34346906 | brown |
| FBXO8 | 26269 | 0.873583749 | 0.35288629 | brown |
| IL17RB | 55540 | 0.87323481 | 0.315545635 | brown |
| FAM189A2 | 9413 | 0.872771128 | 0.313792924 | brown |
| RNF182 | 221687 | 0.872635057 | 0.376946145 | brown |
| PPP1R3D | 5509 | 0.872511403 | 0.663565926 | brown |
| STK17B | 9262 | 0.872290153 | 0.418433489 | brown |
| MCC | 4163 | 0.870860014 | 0.415377449 | brown |
| TRIL | 9865 | 0.870514415 | 0.211308689 | brown |
| IL17RD | 54756 | 0.867780756 | 0.332744583 | brown |
| GNA12 | 2768 | 0.867055073 | 0.311316484 | brown |
| GJB6 | 10804 | 0.866499889 | 0.227168171 | brown |
| CHST7 | 56548 | 0.865826041 | 0.307660354 | brown |
| SELENBP1 | 8991 | 0.865035929 | 0.310472241 | brown |
| CHRDL1 | 91851 | 0.863182953 | 0.462817741 | brown |
| ADCYAP1R1 | 117 | 0.862652071 | 0.36991185 | brown |
| AK022030 /// TROVE2 | 6738 | 0.86170343 | 0.408420561 | brown |
| ARHGAP24 | 83478 | 0.861207057 | 0.439238443 | brown |
| NACC2 | 138151 | 0.860236184 | 0.334441029 | brown |
| SLCO1C1 | 53919 | 0.860235103 | 0.323842448 | brown |
| LHX2 | 9355 | 0.859664083 | 0.306980479 | brown |
| SYT5 | 6861 | 0.920744731 | 0.37578933 | midnightblue |
| SLC17A7 | 57030 | 0.887708717 | 0.452888138 | midnightblue |
| TMEM59L | 25789 | 0.886348726 | 0.431957743 | midnightblue |
| PITPNM2 | 57605 | 0.874301018 | 0.266326439 | midnightblue |
| B9D1 | 27077 | 0.858389615 | 0.343341813 | midnightblue |
| CA11 | 770 | 0.824269421 | 0.475929089 | midnightblue |
| TMEM38A | 79041 | 0.822119685 | 0.338452668 | midnightblue |
| SH3GLB2 | 56904 | 0.819973781 | 0.232147572 | midnightblue |
| CTXN1 | 404217 | 0.818994467 | 0.187934074 | midnightblue |
| NRXN3 | 9369 | 0.818759074 | 0.438407846 | midnightblue |
| CDK5 | 1020 | 0.818722562 | 0.261510011 | midnightblue |
| ARHGDIG | 398 | 0.801818314 | 0.33977235 | midnightblue |
| POU2F1 | 5451 | 0.958621616 | 0.427551834 | PINK |
| ATF7IP | 55729 | 0.898174847 | 0.320461445 | PINK |
| RFX3 | 5991 | 0.874110661 | 0.456315986 | PINK |
| KDM5B | 10765 | 0.874028384 | 0.346949619 | PINK |
| ZNF507 | 22847 | 0.871783867 | 0.452723571 | PINK |
| LSM14A | 26065 | 0.868663639 | 0.410881794 | PINK |
| LCOR | 84458 | 0.867263617 | 0.525332448 | PINK |
| ZNF529 | 57711 | 0.86280051 | 0.33872821 | PINK |
| AHCTF1 | 25909 | 0.860937646 | 0.503162905 | PINK |
| PHIP | 55023 | 0.859790707 | 0.518564877 | PINK |
| SLC35A3 | 23443 | 0.84687741 | 0.467265152 | PINK |
| INTS2 | 57508 | 0.842077758 | 0.493094617 | PINK |
| SMAD2 | 4087 | 0.841744744 | 0.431128371 | PINK |
| AGO3 | 192669 | 0.840616122 | 0.447843614 | PINK |
| SH3GLB1 | 51100 | 0.828618913 | 0.366026562 | PINK |
| HELZ | 9931 | 0.823415678 | 0.356651885 | PINK |
| PURG | 29942 | 0.823319506 | 0.346112764 | PINK |
| RBMS3 | 27303 | 0.820328216 | 0.516531319 | PINK |
| LATS1 | 9113 | 0.819089453 | 0.514000526 | PINK |
| FCHO2 | 115548 | 0.816570514 | 0.36827638 | PINK |
| SNX12 | 29934 | 0.812904524 | 0.471861566 | PINK |

**Supplementary Table 2 The GO analysis of the three modules**

| **Name** | **P-value** | **Adjusted p-value** | **Z-score** | **Combined score** |
| --- | --- | --- | --- | --- |
| Eye development (GO:0001654) | 0.00003227 | 0.01216 | -2.83 | 29.25 |
| Positive regulation of transcription, DNA-templated (GO:0045893) | 0.00004245 | 0.01216 | -5.24 | 52.75 |
| G-protein coupled receptor complex (GO:0097648) | 0.0003421 | 0.003017 | -5.62 | 44.82 |
| Platelet-derived growth factor receptor-ligand complex (GO:1990270) | 0.0003421 | 0.003017 | -5.24 | 41.8 |
| Transmembrane collagen trimer (GO:0030936) | 0.0003421 | 0.003017 | -5.23 | 41.72 |
| SC5b-7 complex (GO:0034995) | 0.0003421 | 0.003017 | -5.22 | 41.68 |
| Pre-T cell receptor complex (GO:0043384) | 0.0003421 | 0.003017 | -5.21 | 41.57 |
| BMP receptor complex (GO:0070724) | 0.0003495 | 0.003017 | -5.48 | 43.65 |
| Interleukin-23 receptor complex (GO:0072536) | 0.0003495 | 0.003017 | -5.45 | 43.37 |
| Spanning component of plasma membrane (GO:0044214) | 0.0003495 | 0.003017 | -5.4 | 42.97 |
| Oncostatin-M receptor complex (GO:0005900) | 0.0003495 | 0.003017 | -5.38 | 42.81 |
| Ciliary neurotrophic factor receptor complex (GO:0070110) | 0.0003532 | 0.003017 | -5.44 | 43.28 |
| Histamine-gated chloride channel complex (GO:0019183) | 0.0003532 | 0.003017 | -5.41 | 43.03 |
| Integral component of external side of plasma membrane (GO:0071575) | 0.0003532 | 0.003017 | -5.41 | 43.03 |
| Interleukin-28 receptor complex (GO:0032002) | 0.0003532 | 0.003017 | -5.41 | 43.01 |
| Interleukin-1 receptor complex (GO:0045323) | 0.0003532 | 0.003017 | -5.35 | 42.56 |
| Ionotropic glutamate receptor complex (GO:0008328) | 0.0003725 | 0.003017 | -5.33 | 42.08 |
| CD40 receptor complex (GO:0035631) | 0.0003804 | 0.003017 | -5.29 | 41.65 |
| Protein import (GO:0017038) | 0.000536 | 0.06634 | -1.32 | 9.94 |
| Phosphatidylinositol binding (GO:0035091) | 0.0005492 | 0.04747 | -3.16 | 23.74 |
| Negative regulation of canonical Wnt signaling pathway (GO:0090090) | 0.000617 | 0.06634 | -3.2 | 23.65 |
| Activating transcription factor binding (GO:0033613) | 0.0006308 | 0.04747 | -2.99 | 22.01 |
| Regulation of cardiac conduction (GO:1903779) | 0.0006362 | 0.06634 | -2.47 | 18.15 |
| Negative regulation of amyloid-beta formation (GO:1902430) | 0.0008001 | 0.06634 | -2.22 | 15.82 |
| Interleukin-17 receptor activity (GO:0030368) | 0.0008001 | 0.04747 | -1.88 | 13.37 |
| Glycosaminoglycan catabolic process (GO:0006027) | 0.0008104 | 0.06634 | -2.37 | 16.88 |
| Retinoid metabolic process (GO:0001523) | 0.0009554 | 0.06843 | -2.37 | 16.49 |
| Glycosaminoglycan metabolic process (GO:0030203) | 0.001136 | 0.0722 | -2.54 | 17.26 |
| Negative regulation of epithelial cell proliferation (GO:0050680) | 0.00126 | 0.0722 | -2.31 | 15.4 |
| Phosphatidylinositol-3,4,5-trisphosphate binding (GO:0005547) | 0.001533 | 0.06823 | -3.15 | 20.44 |
| Nuclear CENP-A containing chromatin (GO:1904834) | 0.001634 | 0.01092 | -1.67 | 10.75 |
| Nucleolar chromatin (GO:0030874) | 0.001634 | 0.01092 | -1.66 | 10.65 |
| Rpd3L-Expanded complex (GO:0070210) | 0.001634 | 0.01092 | -1.64 | 10.55 |
| Set3 complex (GO:0034967) | 0.001634 | 0.01092 | -1.62 | 10.43 |
| Snt2C complex (GO:0070211) | 0.001634 | 0.01092 | -1.61 | 10.32 |
| Perichromatin fibrils (GO:0005726) | 0.001683 | 0.01105 | -1.66 | 10.63 |
| Synaptic vesicle endocytosis (GO:0048488) | 0.002863 | 0.08204 | -0.6 | 3.54 |
| L-glutamate transmembrane transporter activity (GO:0005313) | 0.002863 | 0.1019 | -2.14 | 12.5 |
| Cytokine receptor activity (GO:0004896) | 0.004669 | 0.1224 | -2.45 | 13.17 |
| MiRNA binding (GO:0035198) | 0.005361 | 0.1224 | -2.58 | 13.52 |
| G-protein beta/gamma-subunit complex binding (GO:0031683) | 0.006098 | 0.1224 | -3.23 | 16.47 |
| Transforming growth factor beta binding (GO:0050431) | 0.006877 | 0.1224 | -3.32 | 16.52 |
| Low-density lipoprotein particle receptor binding (GO:0050750) | 0.006877 | 0.1224 | -2.2 | 10.94 |
| R-SMAD binding (GO:0070412) | 0.0077 | 0.1246 | -2.74 | 13.34 |

**Supplementary Table 3 The KEGG pathway analysis of the three modules**

| **Name** | **P-value** | **Adjusted p-value** | **Z-score** | **Combined score** |
| --- | --- | --- | --- | --- |
| Hippo signaling pathway_Homo sapiens_hsa04390 | 0.00001862 | 0.002458 | -1.74 | 18.91 |
| Signaling pathways regulating pluripotency of stem cells_Homo sapiens_hsa04550 | 0.0006647 | 0.04387 | -1.8 | 13.2 |
| Thyroid hormone signaling pathway_Homo sapiens_hsa04919 | 0.001856 | 0.07318 | -1.81 | 11.37 |
| Adherens junction_Homo sapiens_hsa04520 | 0.002217 | 0.07318 | -1.77 | 10.82 |
| Endocytosis_Homo sapiens_hsa04144 | 0.003205 | 0.08461 | -1.86 | 10.7 |
| Cell adhesion molecules (CAMs)_Homo sapiens_hsa04514 | 0.004129 | 0.09083 | -1.61 | 8.81 |
| Mineral absorption_Homo sapiens_hsa04978 | 0.006378 | 0.1203 | -1.54 | 7.76 |

**Supplementary Table 4** Covariance analysis of all key genes (Gender and Age)

| **Hub Gene** | **Age** | **Gender** |
| --- | --- | --- |
| AGT | 0.0628 | 0.3024 |
| AHCYL1 | 0.1186 | 0.6551 |
| APOE | 0.458 | 0.796 |
| ATP1A2 | 0.0646 | 0.5808 |
| CHRDL1 | 0.9277 | 0.95011 |
| EMX2 | 0.0517 | 0.9491 |
| ETNPPL | 0.0564 | 0.4454 |
| FAM189A2 | 0.0191 | 0.8431 |
| FGF2 | 0.08484 | 0.63841 |
| GJA1 | 0.0502 | 0.392 |
| GNA12 | 0.0533 | 0.9372 |
| HEPH | 0.0609 | 0.5363 |
| IL17RB | 0.0884 | 0.9684 |
| MAOA | 0.02272 | 0.51842 |
| METTL7A | 0.058 | 0.4821 |
| NOTCH2 | 0.0819 | 0.5226 |
| NTRK2 | 0.0507 | 0.4599 |
| PARD3 | 0.05713 | 0.84435 |
| PAX6 | 0.3718 | 0.5035 |
| PDLIM5 | 0.01706 | 0.85536 |
| PPAP2B | 0.175 | 0.5207 |
| RAB31 | 0.0533 | 0.3655 |
| SDC2 | 0.3408 | 0.7944 |
| SDC4 | 0.0123 | 0.8018 |
| SLC1A3 | 0.2599 | 0.8043 |
| SMAD2 | 0.4767 | 0.3769 |
| SOX2 | 0.06097 | 0.56252 |
| TP53BP2 | 0.069 | 0.8625 |
| YAP1 | 0.1067 | 0.8436 |
| YES1 | 0.1381 | 0.5475 |

**Supplementary Figure 1** Sample dendrogram and trait heatmap

**
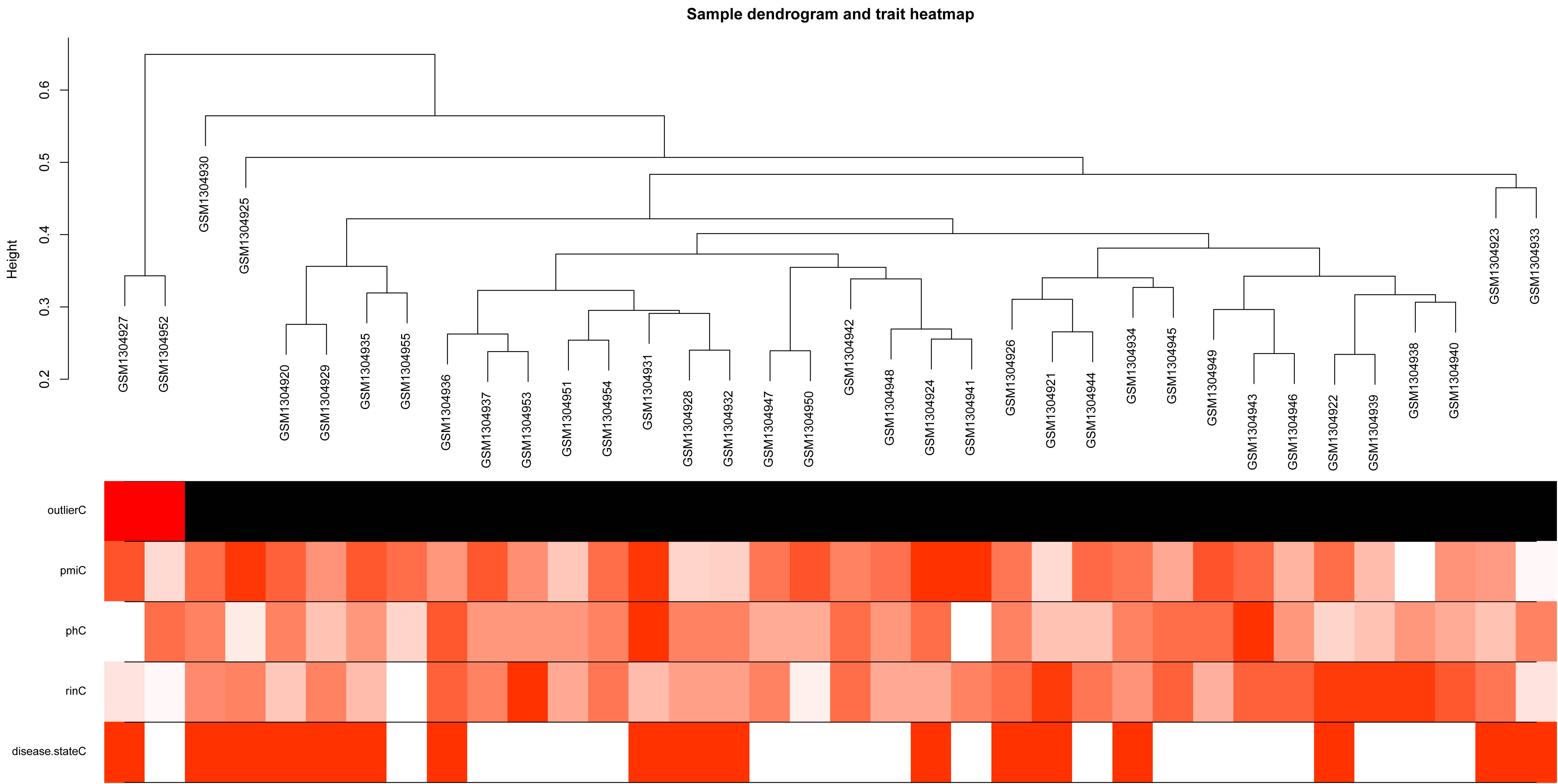
**

**Supplementary Figure 2** Analysis of network topology for various soft-theholding powers

**
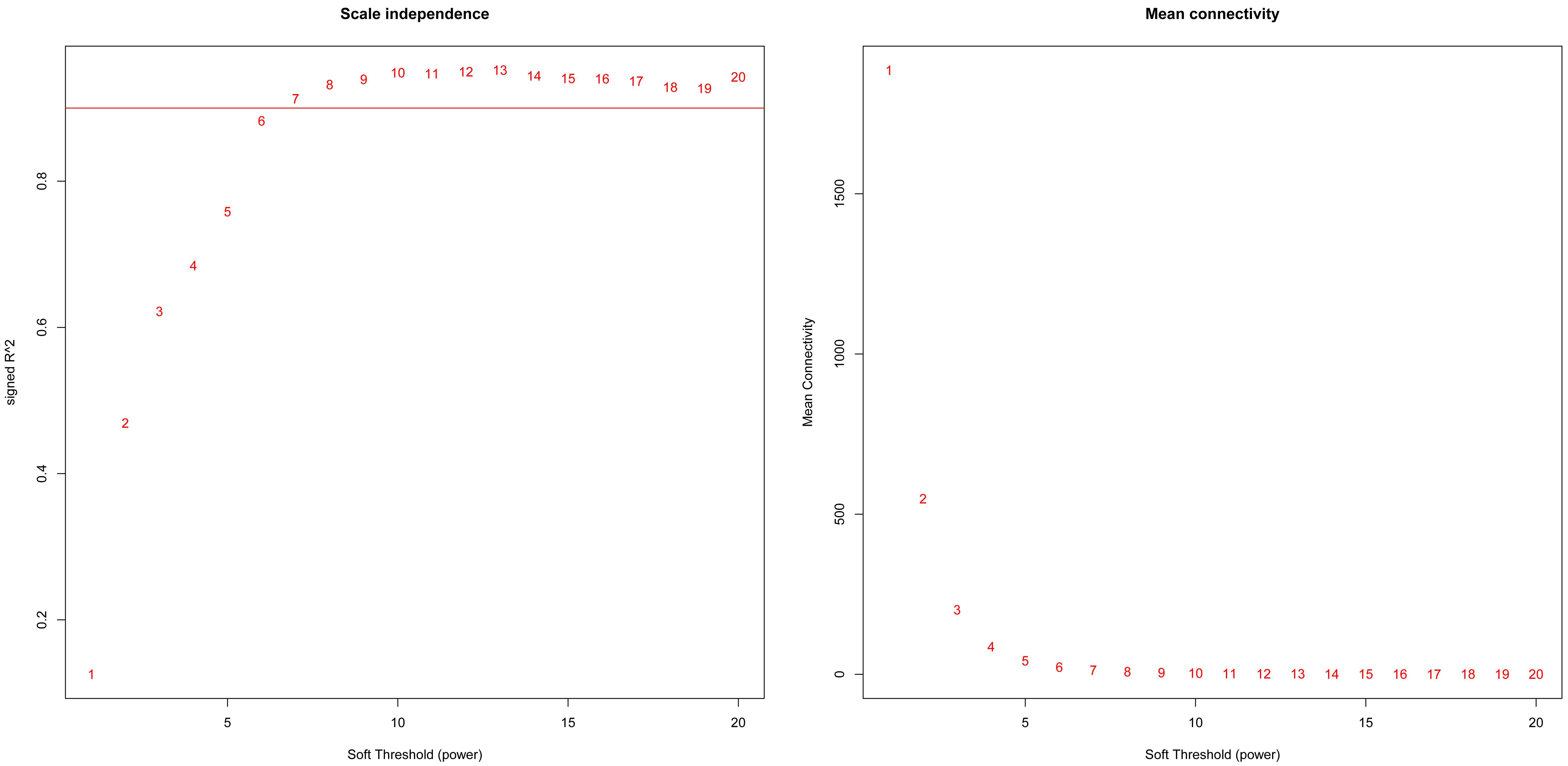
**

**Note:** The left panel showed the scale-free fit index, signed R^2 (y-axis) and the soft threshold power (x-axis).

**Supplementary Figure 3** Clustering dendrogram of genes

**
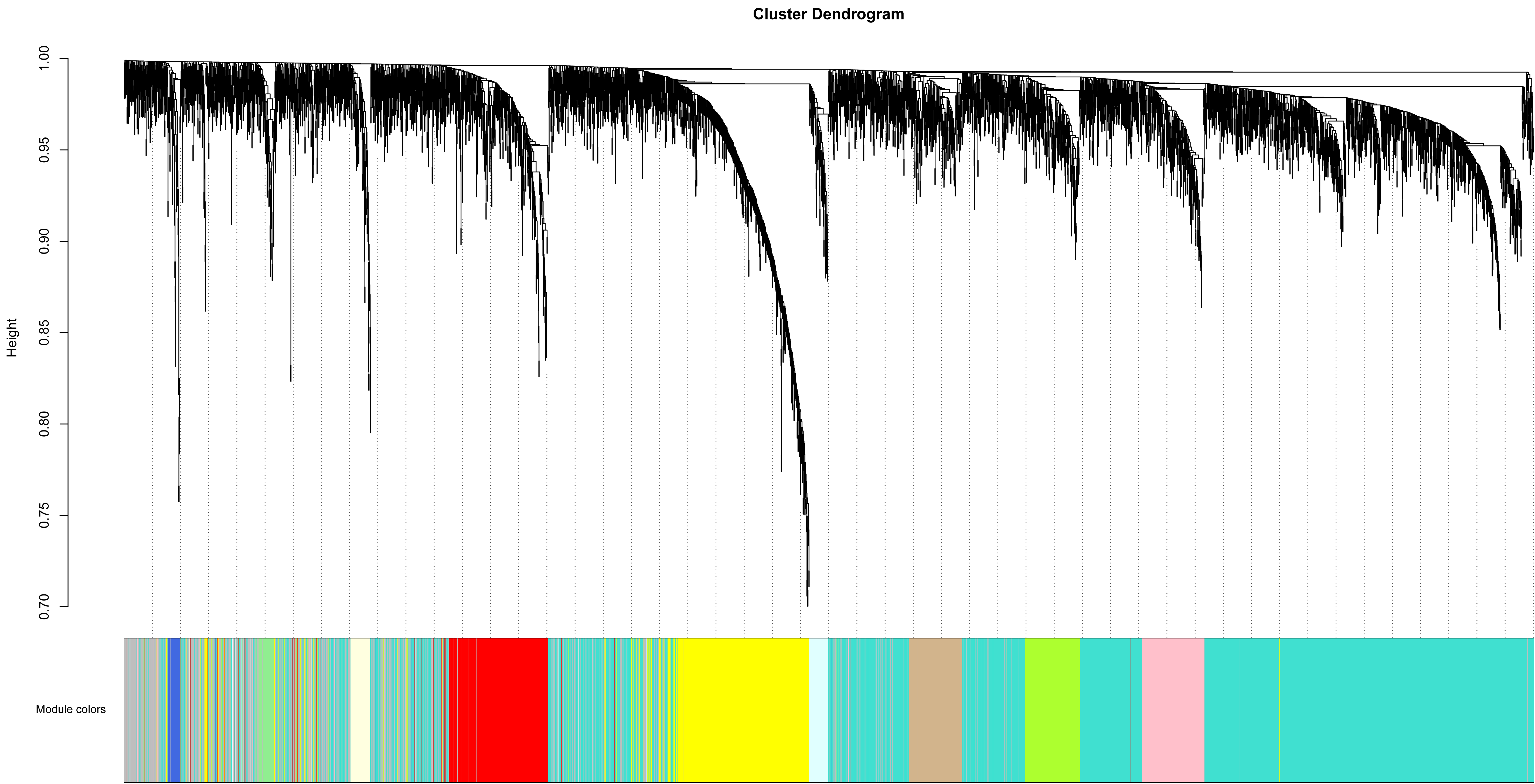
**
